# Downregulation of Mitochondrial TSPO Inhibits Mitophagy and Reduces Enucleation During Human Terminal Erythropoiesis

**DOI:** 10.3390/ijms21239066

**Published:** 2020-11-28

**Authors:** Martina Moras, Claude Hattab, Pedro Gonzalez-Menendez, Suella Martino, Jerome Larghero, Caroline Le Van Kim, Sandrina Kinet, Naomi Taylor, Sophie D. Lefevre, Mariano A. Ostuni

**Affiliations:** 1 Inserm, BIGR, UMR_S1134, Université de Paris, F-75015 Paris, France; martina.moras@inserm.fr (M.M.); chattab@ints.fr (C.H.); suella.martino@inserm.fr (S.M.); caroline.le-van-kim@inserm.fr (C.L.V.K.); sophie.lefevre@inserm.fr (S.D.L.); 2 Institut National de Transfusion Sanguine, F-75015 Paris, France; 3 Institut de Génétique Moléculaire de Montpellier, University of Montpellier, CNRS, F-34293 Montpellier, France; pedro.gonzalez-menendez@igmm.cnrs.fr (P.G.-M.); sandrina.kinet@igmm.cnrs.fr (S.K.); naomi.taylor@igmm.cnrs.fr (N.T.); 4 Laboratoire d’Excellence GR-Ex, F-75015 Paris, France; 5 Unité de Thérapie cellulaire, AP-HP, Hôpital Saint-Louis, F-75010 Paris, France; jerome.larghero@aphp.fr; 6 Pediatric Oncology Branch, Center for Cancer Research (CCR), National Cancer Institute (NCI), National Institutes of Health (NIH), Bethesda, MD 20892, USA

**Keywords:** erythropoiesis, TSPO1, VDAC, mitophagy, PINK1, enucleation

## Abstract

Translocator protein (TSPO) and voltage dependent anion channels (VDAC) are two proteins forming a macromolecular complex in the outer mitochondrial membrane that is involved in pleiotropic functions. Specifically, these proteins were described to regulate the clearance of damaged mitochondria by selective mitophagy in non-erythroid immortalized cell lines. Although it is well established that erythroblast maturation in mammals depends on organelle clearance, less is known about mechanisms regulating this clearance throughout terminal erythropoiesis. Here, we studied the effect of TSPO1 downregulation and the action of Ro5-4864, a drug ligand known to bind to the TSPO/VDAC complex interface, in ex vivo human terminal erythropoiesis. We found that both treatments delay mitochondrial clearance, a process associated with reduced levels of the PINK1 protein, which is a key protein triggering canonical mitophagy. We also observed that TSPO1 downregulation blocks erythroblast maturation at the orthochromatic stage, decreases the enucleation rate, and increases cell death. Interestingly, TSPO1 downregulation does not modify reactive oxygen species (ROS) production nor intracellular adenosine triphosphate (ATP) levels. Ro5-4864 treatment recapitulates these phenotypes, strongly suggesting an active role of the TSPO/VDAC complex in selective mitophagy throughout human erythropoiesis. The present study links the function of the TSPO/VDAC complex to the PINK1/Parkin-dependent mitophagy induction during terminal erythropoiesis, leading to the proper completion of erythroid maturation.

## 1. Introduction

Erythropoiesis is a multistep differentiation process that gives rise to mature erythrocytes. Hematological stem cells first proliferate and commit into the erythroid lineage, giving rise to the burst forming unit-erythroid (BFU-E) and colony forming unit-erythroid CFU-E progenitors. Upon the second phase, also called terminal differentiation, CFU-E will successively differentiate proerythroblast (Pro-E), early and late basophilic erythroblasts (Baso-E), polychromatic erythroblast (Poly-E), and orthochromatic erythroblast (Ortho-E). Mammalian erythroblasts expel the nuclei from Ortho-E stage, producing the reticulocyte and the pyrenocyte that will be engulfed by macrophages of the erythroblastic island. 

During the end of the terminal maturation, erythroblasts lose all their organelles, such as the Golgi apparatus, endoplasmic reticulum (ER), ribosomes, and mitochondria (reviewed in [1]). This elimination can occur both by unspecific or by selective macro-autophagy. In both, activation of autophagy-related proteins (ATG) leads to the formation of the phagophore, a membrane structure originated from ER, that surrounds cytoplasmic cargos forming the autophagosome. Then this late migrates and fuses to lysosomes, where cargos are degraded [2,3]. In selective macroautophagy, targets are specific cell components that should be eliminated, such as mitochondria. Although bulk and selective macroautophagy share the same core machinery, this later involves specific proteins exposed at the surface of targeted organelles as a signal to recruit the phagophore [4]. 

Although some proteins from the autophagic machinery (Ulk1, Atg4, Atg7) were shown to be essential for organelle removal in erythroid precursors, most of these studies were performed in human late reticulocytes or in ATG knock-out mice [5,6,7,8,9,10,11,12,13] and, up to now, there are not enough data studying organelle clearance at early erythroblast stages. Interestingly, the outer mitochondrial membrane (OMM) protein NIX, was first identified in mice to be involved on mitophagy induction at the reticulocyte stage through direct interaction with LC3, which is a well characterized connector protein, bridging the targeted organelle to the phagophore membrane before engulfment into the autophagosome [10,11,12].

In non-erythroid cells, the best characterized mitophagy pathway is the PTEN-induced kinase-1 (PINK1)/Parkin pathway. In healthy functional mitochondria, PINK1 is addressed to the OMM, translocated to the inner membrane, where it is cleaved by PARL, allowing its retrotranslocation to the cytoplasm and its degradation by the proteasome. However, on defective mitochondria (i.e., low membrane potential), there is an accumulation of the uncleaved form of PINK1 at the OMM, which results in the recruitment of Parkin, an E3 ubiquitin ligase, and the subsequent clearance of the damaged mitochondria by mitophagy [7,14,15]. VDAC (voltage-dependent anion channel), one of the most abundant OMM proteins, is a target for Parkin-mediated ubiquitination. This process is partially modulated by its protein partner, the translocator protein (TSPO), which modulates reactive oxygen species (ROS) levels interfering with VDAC ubiquitination [16]. VDAC is also involved in the proper recruitment of Parkin to defective mitochondria [16,17,18]. 

Surprisingly, this mitophagy pathway was never studied in erythroblast maturation. We then aim to determine whether TSPO is able to modulate mitophagy throughout human erythropoiesis and whether this modulation involves VDAC/TSPO interaction. We first tested the effect of genetic downregulation of TSPO1. We observed that reduction on TSPO1 levels is associated with a strongly diminished enucleation rate and induction of apoptosis. Moreover, we observed a significantly higher mitochondria content at later differentiation stages and a strong reduction of PINK1 protein, but we did not observe any change in NIX protein. We have then used a pharmacological approach using Ro5-4864 (4′-Chlorodiazepam), a benzodiazepine derivative of diazepam that lacks affinity for GABAA receptors [19] and shows high affinity for TSPO1. This ligand requires the presence of other TSPO1 partners like VDAC [20], and is thought to bind at the VDAC/TSPO1 interface [21], modulating the function of VDAC as well [22]. Used on differentiating erythroblasts, Ro5-4864 treatment also reduces enucleation and induces apoptosis and mitochondria retention as observed upon TSPO1 invalidation. Thus, these findings demonstrate the role of the complex VDAC/TSPO1 in enucleation and in mitochondrial clearance driven by the PINK1/Parkin pathway throughout human erythroblast differentiation. Moreover, pharmacological modulation of the TSPO/VDAC complex could be explored as a potential therapeutic tool to improve organelle clearance upon dyserythropoiesis.

## 2. Results

### 2.1. Downregulation of TSPO1 does not Affect Differentiation Kinetics but Reduces Erythroblast Enucleation

**Figure 1:**
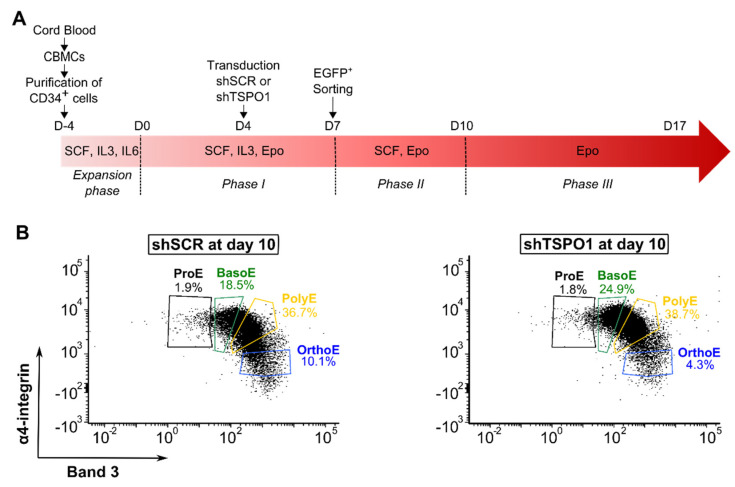

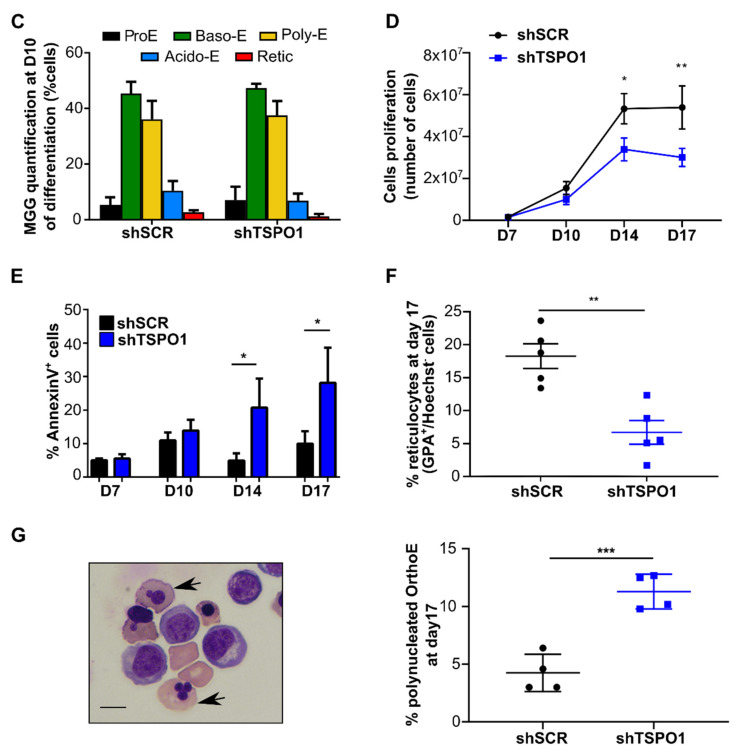
TSPO1 downregulation does not affect differentiation kinetics but diminished enucleation rate. (**A**) Schematic of the ex vivo erythroid differentiation protocol following shRNA-mediated downregulation of TSPO1 (CBMC, cord blood mononucleated cells; shSCR, scramble shRNA; shTSPO1, TSPO1 shRNA). CD34+ progenitors were isolated from cord blood and transduced at Day 4 with a lentiviral vector harboring either the TSPO1 shRNA or scramble shRNA, together with the green fluorescent protein (EGFP) transgene. EGFP+ cells were sorted at Day 7 and differentiated until Day 17. (**B**) Representative profile in flow cytometry of cells at Day 10 of erythropoiesis in scramble shRNA and TSPO1 shRNA. Glycophorin A positive (GPA+) cells are gated according to α4-integrin and Band 3 expression levels to discriminate the different stages of differentiation. (**C**) May-Grünwald Giemsa (MGG)-based quantification at Day 10 of erythroid differentiation. (**D**) Erythroblast proliferation at Day 7, 10, 14, and 17 (*n* = 4). (**E**) Apoptosis assay (annexin V) by flow cytometry at Day 7, Day 10, Day 14, and Day 17 (*n* = 3). (**F**) Enucleated cells (GPA+/Hoechst- cells) were quantified by flow cytometry at Day 17 of differentiation (*n* = 5). (**G**) Representative images (left) and quantification (right) of polynucleated Ortho-E (arrow) at Day 17 after MGG coloration (*n* = 3). Scale bar = 20 μm. * *p* < 0.05, ** *p* < 0.01, *** *p* < 0.001.

To evaluate the role of mitochondrial TSPO1 in mitophagy during erythroid differentiation, we have downregulated TSPO1 expression using an shRNA approach. We transduced cells at Day 4 with lentiviral vectors containing a short hairpin RNA scramble (shSCR) or a short hairpin RNA targeting TSPO1 (shTSPO1) before EGFP fluorescence-based sorting at Day 7, as shown in Figure 1A. Knockdown efficiency was assessed at Day 10 of erythroid differentiation. A strong downregulation was observed at both mRNA (88.6% ± 1.9) and protein (91.6% ± 3.1) levels, as shown in Figure S1.

The effect of TSPO1 downregulation on the erythroblast’s maturation was evaluated by flow cytometry on GPA^+^ cells gated according to the expression of the surface markers α4-integrin and Band 3 [23]. No differences were observed between shTSPO1-transduced and control cells, as shown in Figure 1B. We compared this result to the quantification analysis based on May-Grünwald Giemsa (MGG) staining and confirm the absence of differences upon erythroid maturation, as shown in Figure 1C. At the end of the differentiation, however, a decrease in the absolute cell number was observed, as shown in Figure 1D, concomitantly with an increase in the percentage of annexin V-positive cells, as shown in Figure 1E. 

At Day 17, the percentage of reticulocytes was analyzed by flow cytometry (GPA^+^, Hoechst^−^) showing a significant 2-fold decrease of the enucleation rate in shTSPO1-transduced cells, as shown in Figure 1F. Increased apoptosis induction occurs when cell cultures reach the orthochromatic stage, confirming that the impairment of enucleation has a dramatic outcome on erythroblast viability. By MGG staining, we observed an increase in the percentage of polylobulated orthochromatic erythroblasts in shTSPO1-transduced cells, as shown in Figure 1G, suggesting that enucleation-related cytokinesis might be impaired by TSPO1 downregulation after the stage of nucleus partition but before the cytoplasm scission. 

### 2.2. TSPO1 Downregulation Affects Mitochondrial Clearance

**Figure 2:**
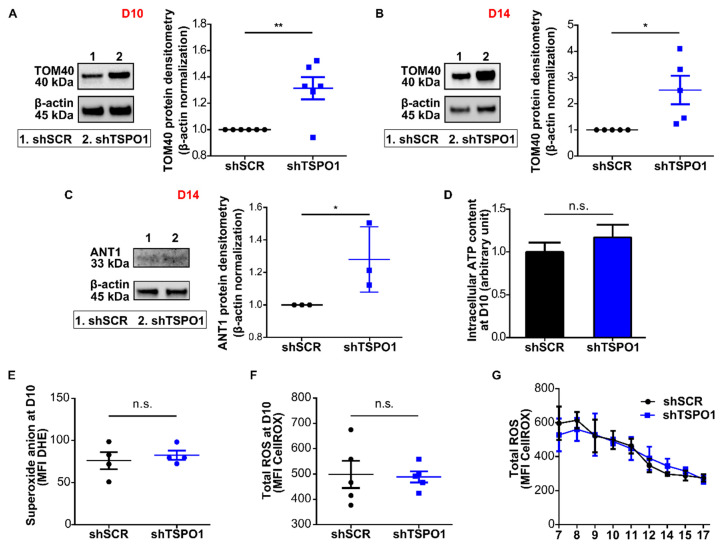
TSPO1 downregulation causes mitochondrial retention. Western blot quantification of TOM40 as mitochondrial mass reporter at (**A**) Day 10 (*n* = 6) and (**B**) Day 14 of human erythroid differentiation (*n* = 5). (**C**) Western blot quantification of adenine nucleotide transporter (ANT) as mitochondrial mass reporter at Day 14 (*n* = 3). Levels in shSCR-transduced cells were arbitrarily set at “1”. * *p* < 0.05. (**D**) Intracellular adenosine triphosphate (ATP) quantification by luminometry (*n* = 3) at Day 10. Level in shSCR-transduced cell was arbitrarily set at “1”. (**E**) Superoxide anion quantification measured at Day 10 of differentiation by flow cytometry (DHE) (*n* = 4). (**F**) Total reactive oxygen species (ROS) production quantification measured at Day 10 of differentiation by flow cytometry (CellROX) (*n* = 5) at Day 10 and (**G**) all along the differentiation. * *p* < 0.05, ** *p* < 0.01, n.s. not statistically significant

Based on previous results in non-erythroid cell lines supporting a role of TSPO1 in mitophagy [16], we hypothesized that TSPO1 downregulation could inhibit mitochondrial clearance disturbing terminal erythroid differentiation as well. We analyzed the mitochondrial mass by Western blot using the OMM protein TOM40 and the inner mitochondrial membrane (IMM) protein adenine nucleotide transporter (ANT1) as mitochondrial markers. A significant increase was observed at both Day 10, as shown in Figure 2A, and Day 14, as shown in Figure 2B,C. This suggests that the downregulation of TSPO1 caused a defect in mitochondria clearance. We then tested whether this mitochondria retention could affect mitochondria functionality. With respect to the total mitochondrial mass, no differences were observed in adenosine triphosphate (ATP) content, as shown in Figure 2D, or ROS levels, as shown in Figure 2E–G, comparing shTSPO1- with shSCR-transduced cells during differentiation.

### 2.3. TSPO1 Downregulation Affects Mitophagy

**Figure 3:**
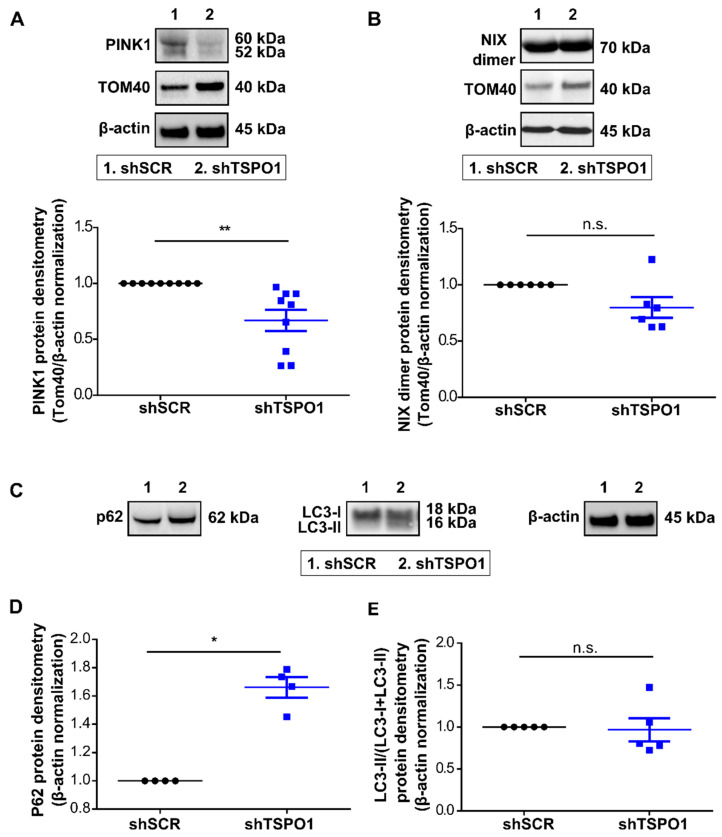
TSPO1 downregulation diminishes mitophagy-related protein levels. Western blot-based quantification of (**A**) PINK1 (*n* = 9), (**B**) NIX (*n* = 6), at Day 10 of differentiation normalized by quantity of mitochondria (TOM40) on total number of cells (β-actin). Representative images (**C**) and Western blot-based quantification of (**D**) p62 and (**E**) LC3-II/LC3-I+LC3-II ratio. Levels in shSCR-transduced cells were arbitrarily set at “1”. * *p* < 0.05, ** *p* < 0.01, n.s. not statistically significant.

To evaluate whether the mitochondria accumulation observed was due to an impairment in the autophagic process and in particular in the canonical PINK1/Parkin pathway, we studied the upstream signal, i.e., the accumulation of PINK1 and NIX at the OMM. In shTSPO1-transduced cells, we observed a decrease of PINK1 protein level when normalized to TOM40, as shown in Figure 3A. We have then analyzed the alternative NIX-driven mitophagy pathway. The levels of NIX protein were not significantly modified by shTSPO1 transduction, as shown in Figure 3B. Together, these data suggest that TSPO1 downregulation causes an accumulation of mitochondria due to an impairment of mitophagy induction through the PINK1/Parkin pathway, without affecting the NIX pathway. To assess whether general autophagy was also affected by TSPO1 knockdown, we have quantified LC3 activation and the levels of p62, an adaptor protein that allows bridging between the ubiquitinated cargos and the phagophore membrane [24]. While there was a significant accumulation of p62, the autophagy-mediated lipidation of LC3-I to LC3-II in the autophagosome membrane was not altered, as shown in Figure 3C,D.

### 2.4. Ro5-4864 Recapitulates the Phenotypes of TSPO1 ShRNA-Mediated Downregulation

**Figure 4:**
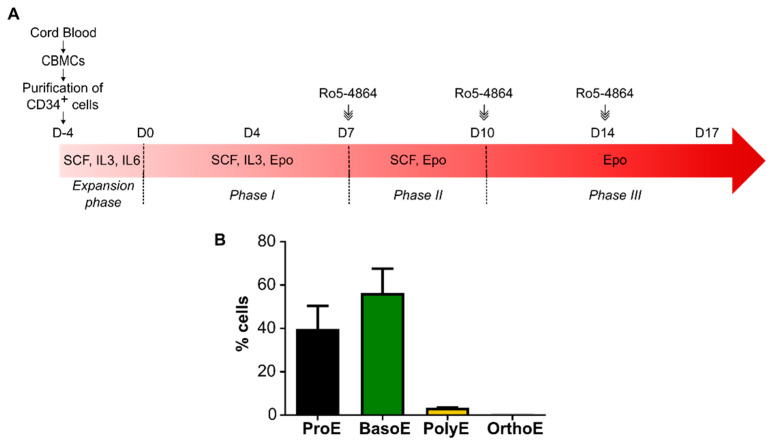
Ex vivo erythropoiesis protocol adding Ro5-4864 at the terminal phase. (**A**) Schematic of the ex vivo erythroid differentiation protocol following Ro5-4864 treatment. Cord blood mononucleated cells (CBMCs) were used to isolate CD34+ progenitors. Fifty micromolar of Ro5-4864 were added starting from Day 7. At Day 10 and 14, when media was changed, Ro5-4864 was added at the same concentration. (**B**) Quantification of the percentage of the different erythroblastic stages (ProE = proerythroblast, BasoE = basophilic erythroblast, PolyE = polychromatic erythroblast, OrthoE = orthochromatic erythroblast) after MGG coloration at Day 7 of differentiation (*n* = 3).

To better understand whether the effect of TSPO1 downregulation is modulated by its partner VDAC, we performed an ex vivo erythroid differentiation in the presence of the drug ligand Ro5-4864, known to bind at the TSPO1/VDAC interface. We previously reported that 50 µM of Ro5-4864 is optimal to modulate several functions of the complex [22,25]. To target the terminal phase of the differentiation, the ligand was added from Day 7, as shown in Figure 4A, when cells are mostly at Pro-E and Baso-E stages, as shown in Figure 4B. At Day 10 and 14, when media was changed, the ligand was maintained in the culture.

**Figure 5:**
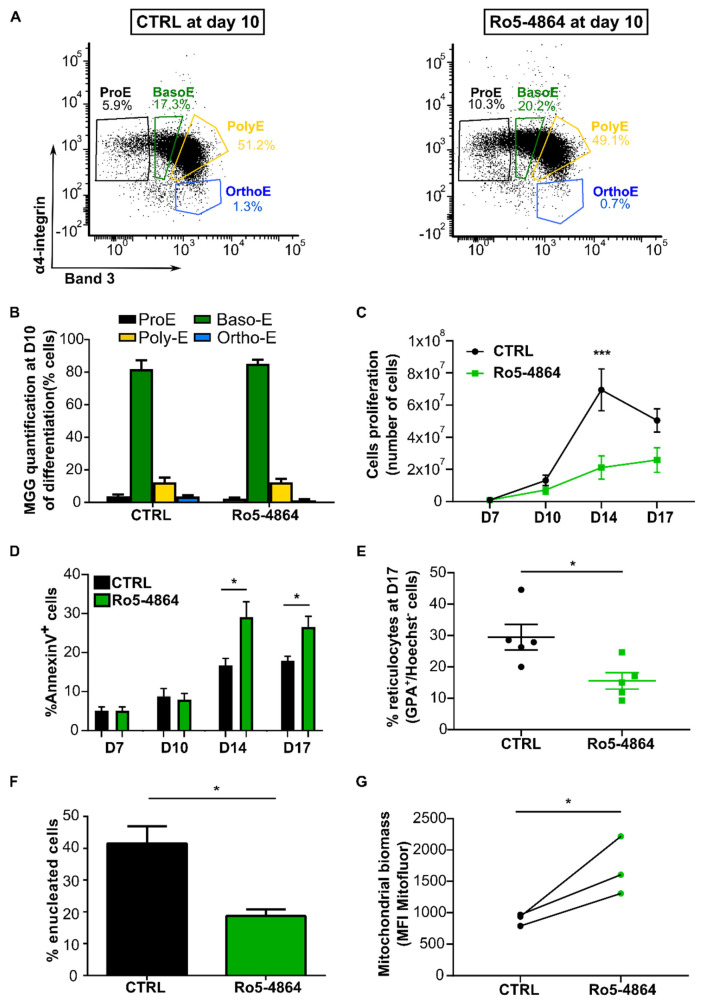
Ro5-4864 recapitulates the phenotypes of TSPO1 shRNA-mediated downregulation. (**A**) Representative profile in flow cytometry of control cells (CTRL) and Ro5-4864 treated cells during terminal erythropoiesis at Day 10. GPA+ cells are gated according to α4-integrin and Band 3 expression levels to discriminate the different stages of differentiation. (**B**) MGG-based quantification. (**C**) Erythroblast proliferation at Day 7, 10, 14, and 17 (*n* = 4). (**D**) Apoptosis assay (annexin V) by flow cytometry at Day 7, Day 10, Day 14, and Day 17 (*n* = 4). (**E**) Enucleated cells (GPA+, Hoechst- cells) were quantified by flow cytometry at Day 17 of differentiation (*n* = 5) and (**F**) counted after MGG coloration the same day (*n* = 3). (**G**) Mitochondrial content of erythroblasts at Day 10 of differentiation detected by flow cytometry (MitoFluor) (*n* = 3). * *p* < 0.05, and *** *p* < 0.005.

After the addition of the ligand at Day 7, we analyzed the differentiation kinetics of control cells (CTRL, in the presence of 0.25% ethanol) compared to Ro5-4864 treated cells by flow cytometry. As observed for shTSPO1-transduced cells, no differences were observed between cells treated with Ro5-4864 compared to control ones along the terminal phase of the differentiation (Day 7, 10, 14, 17), as shown in Figure 5A. This was confirmed by MGG analysis, as shown in Figure 5B. However, we observed a significant decrease in the absolute cell number at Day 14, as shown in Figure 5C, together with an increase in the percentage of annexin V-positive cells in Ro5-4864 treated cells, as shown in Figure 5D. A significant decrease of half of the enucleation rate was observed after Ro5-4864 treatment, as shown in Figure 5E. This data was confirmed by the quantification of reticulocyte after MGG coloration at Day 17 where we observed a decrease of half in reticulocyte quantification in Ro5-4864 treated compared to control cells, as shown in Figure 5F. These results suggest that, as TSPO1 downregulation by shRNA, Ro5-4864 does not cause any alteration in the differentiation kinetics but impairs Ortho-E enucleation in human terminal erythropoiesis.

In the non-erythroid cell line, it was demonstrated that the complex VDAC/TSPO1 played a role in the modulation of mitophagy [16]. To evaluate whether the defect in mitochondrial clearance observed upon shTSPO1 transduction was dependent on the TSPO1/VDAC complex, we quantified by flow cytometry the mitochondrial biomass at Day 10 of differentiation in CTRL and Ro5-4864 treated cells using MitoFluor. A statistically significant increase was observed in mitochondrial biomass in the whole cell population at Day 10 of differentiation, as shown in Figure 5G.

## 3. Discussion

Several studies have shown upon erythroid differentiation, clearance of mitochondria occurs at the reticulocyte stage by mitophagy [9,10,11]. However, as these studies were mostly performed on mice, it is still unclear the mechanisms occurring during human erythroblast maturation. As the role of the TSPO/VDAC complex on the mitochondrial clearance by non-erythroid cell lines was previously demonstrated [16,17,18], we aimed to determine whether this complex is involved in mitophagy throughout human erythropoiesis by knocking down TSPO1 and by using the synthetic drug ligand Ro5-4864, a TSPO ligand known to bind at the interface of TSPO1/VDAC.

We first used a shRNA approach to repress TSPO1 expression during the terminal erythropoiesis. We observed no difference in the kinetics of erythroid differentiation monitored by both the quantification after MGG coloration and the flow cytometry-based Band 3/α4-integrin expression. Nevertheless, the enucleation process was strongly impaired, and we observed the presence of high numbers of polylobate Ortho-E. This feature would suggest a deregulation of the scission of the cytoplasm after the stage of nucleus partition, favoring the presence of polylobate cells at the end of the differentiation. Moreover, TSPO1 downregulation induced an increase in apoptosis at Day 14 and 17, suggesting that the impairment in the enucleation could induce an increase in cell death.

We have then studied the effect of downregulating TSPO1 on mitochondrial clearance and mitophagy pathways. The reduction of TSPO1 expression causes the retention of mitochondria associated with altered PINK1 accumulation but without affecting the NIX pathway. This strongly supports that TSPO1 that plays a crucial role in mitochondrial clearance during human erythropoiesis, affecting the accumulation of PINK1 at the OMM, and causing a decrease in the recruitment of the autophagosome membrane to mitochondria to be eliminated.

As mentioned above, TSPO is known to be associated with VDAC in mitochondrial membrane complex [26,27,28] and is able to modulate VDAC-dependent mitophagy in non-erythroid cells [16]. Moreover, we have previously demonstrated that some TSPO ligands could modulate the activity of the TSPO/VDAC complex [22]. We have then treated erythroblasts with Ro5-4864, a well characterized TSPO ligand, which is described to bind to the TSPO/VDAC interface [21].

Addition of Ro5-4864 does not cause any alteration of the kinetics of expression of canonical markers of the erythroid differentiation, suggesting that none of the functions of the complex modulated by the use of the ligand alters the kinetics of human erythroid differentiation. We observed, however, a decrease in the proliferation rate for TSPO1 downregulation. This data may suggest that modulating the TSPO1/VDAC function with Ro5-4864 could cause a decrease in the proliferation capacity of maturing erythroblasts. 

We have also observed a diminished enucleation rate and impaired decrease in mitochondrial biomass when Ro5-4864 was added in the culture, suggesting an effect of the complex in the enucleation and mitophagy mechanisms. 

The blockage of enucleation could be due to a TSPO1 function in cholesterol transport, alone or as partner on the complex VDAC/TSPO1 [28,29,30]. Moreover, recent studies have shown the importance of cholesterol during the enucleation process [31], in particular demonstrating that a depletion in cholesterol could affect the cytokinesis [32]. Those results support the hypothesis that a defect in this transport may cause a modification in the cholesterol distribution within cell membranes, altering membrane fluidity and causing the defect in the cytokinesis.

Altogether, our results strongly support the hypothesis that the complex composed by VDAC and TSPO1 is involved in different functions during terminal erythropoiesis, as modulation of enucleation and regulation of the selective autophagy of mitochondria driven by the PINK1/Parkin pathway. Moreover, as differentiation could be modulated by the drug ligand Ro5-4864, these results open new perspectives concerning the use of alternative pharmacological tools to modulate erythroid differentiation in the case of different diseases involving dyserythropoiesis.

Notably, we have recently demonstrated that some members of a new family of TSPO synthetic ligands are able to reduce the cholesterol uptake by the erythrocyte [25] and modulate VDAC oligomerization at the erythrocyte plasma membrane [22].

Finally, it could be interesting to explore the possible role of the isoform TSPO2, which was previously characterized as an erythroid-specific isoform [33], mainly expressed at the last erythroblast stages [34] and localized at the ER/nuclear envelope [33,35] but also at the erythrocyte plasma membrane [36].

## 4. Materials and Methods

### 4.1. Purification and Culture of CD34^+^ Cells

Cord blood mononuclear cells (CBMCs) from healthy donors were used in this study. Umbilical cord blood that cannot be qualified for use in patients was obtained from the center for biological resources from Saint-Louis Hospital Cord Blood Bank registered to the French Ministry of Research under number AC-2016-2756, and to the French Normalization Agency under number 201/51848.1. This study was approved and conducted according to institutional ethical guidelines of the National Institute for Blood Transfusion (N°2019-1, INTS, Paris, France). It was also approved by the ethics committee of the INSPIRE program (Horizon 2020 grant agreement 665850, November 2017. All procedures were carried out in accordance with the Declaration of Helsinki. Written informed consent was given by the donors. CBMCs were separated from whole blood using Ficoll (GE healthcare) and CD34^+^ cells were purified from cord blood by positive selection using the magnetic-activated cell sorting magnetic beads system (Miltenyi Biotec, Paris, France), according to the manufacturer’s instructions.

CD34^+^ cells were cultured in Iscove’s Modified Dulbecco’s Medium (Invitrogen), 2% human peripheral blood plasma (Stem Cell Technologies, Cambridge, UK), 3% human AB serum (Sigma Aldrich, St Quentin Fallavier, France), 15% BIT 9500 Serum Substitute (Stem Cell Technologies), and 3 IU/mL heparin (Sigma Aldrich). For the expansion phase (Day -4 to Day 0), cells were seeded at a concentration of 10^5^ cells/mL in culture medium supplemented with 25 ng/mL stem cell factor (SCF, Miltenyi Biotec), 10 ng/mL IL-3 (Miltenyi Biotec), and 10 ng/mL IL-6 (Miltenyi Biotec). In Phase I (Day 0 to Day 6), medium was supplemented with 10 ng/mL SCF, 1 ng/mL IL-3 (Miltenyi Biotec), and 3 IU/mL erythropoietin. IL-6 was omitted from the culture medium. In Phase II (Day 7 to Day 10), IL-3 was omitted from the culture medium. In Phase III (that lasted until Day 17), SCF was omitted. Then, 50 μM Ro5-4864 (Sigma Aldrich), solubilized in 0.25% ethanol, was added at Day 7, 10, and 14.

### 4.2. TSPO1 Silencing Plasmid and Lentiviral Production 

Lentivirus particles were produced by co-transfection of HEK293T cells with the plasmids MISSION^®^ pLKO.1-puro Non-Target shRNA Control Plasmid DNA (TRCN0000297481) or shRNA anti-TSPO1 (TRCN0000297481, responding sequence, 5′-GCAGTTGGCTACAAGACTGAT-3′) with the 714 bp EGFP sequence that was inserted in place of the puromycin gene at the unique BamHI and KpnI restriction sites, together with the helper plasmids Δ8.9 and VSV-G in Hank’s buffer saline (HBS) buffer (454 μM NaCl, 23 mM Na_2_HPO_4_, 28 mM KCl, 216 mM dextrose, pH = 7.05) and 132 mM CaCl_2_. On the following day, the medium was replaced with Dulbecco’s modified eagle’s medium (DMEM) without serum, and the cells were cultured for 1 day before the virus was collected. Viruses were concentrated by ultracentrifugation for 90 min at 22,000 rpm at 4 °C.

### 4.3. Transduction of CD34^+^ Progenitors

Cells undergoing erythroid differentiation were transduced at Day 4 with lentiviral particles containing a shRNA scramble (shSCR) or a shRNA targeting TSPO1 (shTSPO1) as described in the previous section, at a multiplicity of infection (MOI) of 10. Transduction efficiency was evaluated by the percentage of EGFP+ cells and EGFP+ cells were sorted at Day 7 of erythroid differentiation using the SONY SH800 cell sorter.

### 4.4. Antibodies and Dyes 

The following antibodies (Ab) and corresponding dilution were used for Western blotting: monoclonal mouse anti-TSPO1 Ab (ab109497, 1:10,000, Abcam, Cambridge, UK), rabbit anti-MAP LC3B Ab (Sigma Aldrich, L7543, 1:200), rabbit anti-p62 Ab (GTX100685, 1:1000, GeneTex, Alton Pkwy Irvine, CA, USA), rabbit anti-PINK1 (GTX107851, 1:1000, GeneTex, Alton Pkwy Irvine, CA, USA), rabbit anti-BNIP3L (GTX111876, 1:1000, GeneTex, Alton Pkwy Irvine, CA, USA), mouse anti-TOM40 Ab (sc-365467, 1:1000, Santa Cruz Biotechnology, Dallas, TX, USA), rabbit anti-ANT polyclonal antibody (a generous gift from Dr G. Brandolin, dilution 1:1000 [37]), HRP-coupled anti-rabbit Ab (111-035-144, 1:5000, Jackson Immuno Research Europe, Cambridgeshire, UK), HRP-coupled anti-mouse (Jackson, 115-035-003, 1:5000), HRP-coupled anti-actin Ab (13E5, 1:3000, Cell signaling, Leiden, The Netherlands). PE-conjugated mouse anti-human Band 3 (IBGRL, 9439PE BRIC 6, 1:50), PE-Cy7-conjugated mouse anti-human CD235a (BD Pharmigen™, 563666, 1:20, Becton Dickinson, Rungis, France), APC-H7-conjugated mouse anti-human CD49d (BD Pharmigen™, 656153, 1:10) were used for flow cytometry. Mitochondrial mass was assayed using MitoFluor™ Red 589 (M-22424, 250 nM, Thermo Fisher Scientific Dardilly, France). Hoechst 34,580 (BD Pharmigen™, 565877, 1 µg/mL) was used to label nucleus. PE-coupled Annexin V (BD Pharmingen™, 559763, 1:20) and 7-Aminoactinomycin D (7AAD, Thermo Fisher Scientific, A1310, 1:200) were used for apoptosis and viability assay in flow cytometry, respectively. 

### 4.5. Flow Cytometry-Based Analysis of Erythroid Differentiation

Cells were analyzed for surface marker expression, mitochondrial content, and the presence of a nucleus, starting at Day 7. Briefly, 10^5^ cells were stained with MitoFluor and Hoechst 34,580 in media for 30 min at 37 °C. Cells were washed and subsequently stained with fluorochrome-conjugated antibodies against glycophorin A (GPA), Band 3 and α4-integrin in phosphate buffer saline (PBS) 2% bovine serum albumin (BSA), for 30 min at 4 °C. Cells were washed once with PBS and 7-AAD was added prior to acquisition. Cells were analyzed using a BD FACSCanto™ (Becton Dickinson, Rungis, France), and data were acquired with Diva software (Diva 9.0, Becton Dickinson, Rungis, France) and analyzed using FCS express 6 Flow Research Edition software (FCS 6.0, De Novo Software, Pasadena, CA, USA).

### 4.6. MGG-Based Identification of Erythroblast Stages

Overall, 10^5^ of total and sorted cells according to α4-integrin and Band 3 surface expression as previously described [23] were cytospun using the Thermo Scientific Shandon 4 Cytospin to validate the sorting gates. The slides were stained with May-Grünwald (Sigma Aldrich, MG500, St Quentin Fallavier, France) solution for 5 min, with May-Grünwald solution diluted by half for another 5 min, and subsequently stained with Giemsa solution (Sigma Aldrich, GS500, St Quentin Fallavier, France) diluted 10 times for 15 min. Cells were imaged using a Nikon Eclipse Ti-S inverted microscope with a 40 × /0.6 Plan Fluor objective.

### 4.7. Apoptosis Assay 

Cells were washed in PBS and then resuspended in 100 μL of annexin buffer containing PE-conjugated annexin V. After 15 min of incubation at room temperature in the dark, 400 μL of annexin buffer was added. Viability was assessed by flow cytometry V10.7.1 (BD FACSCanto™, Becton Dickinson, Rungis, France).

### 4.8. SDS-PAGE and Immunoblotting

Whole-cell lysates of cultured cells were prepared in 10 mM Tris-HCl pH 6.8, 1 mM EDTA, 10% SDS buffer, containing 1X cOmplete™ protease cocktail inhibitor (Merck 11873580001, Darmstadt, Germany). After sonication, cells were frozen for at least 1 h. Protein quantification was performed by Pierce™ BCA Protein Assay Kit (Thermo Fisher Scientific, Dardilly, France) and 30 μg of proteins were separated using NuPAGE® 4–12% Bis-Tris gels. After protein transfer to a nitrocellulose membrane using the Trans-Blot Turbo Transfer System (Bio-Rad), immunoblotting was done with a primary antibody overnight at 4 °C. Proteins of interest were revealed using HRP-conjugated secondary antibodies and the ECL™ Western Blotting Reagents kit (Merck, GE Healthcare, RPN2106). Images were acquired by ChemiDoc™ Imaging Systems (Bio-Rad, Marne la Coquette, France) and band intensity was quantified with Image Lab software 6.0 (Bio-Rad).

### 4.9. Quantitative Real Time QPCR

Total RNA was extracted using RNEasy kit (Qiagen, Les Ulis, France) and quantified by NanoDrop 2000c (Thermo Scientific™). Reverse transcription of 2 μg of total RNA was performed using MultiscribeTM Reverse Transcriptase (Applied Biosystems, Thermo Fisher, Dardilly, France) and gene expression assays were performed using QuantiNova SYBR Green RT-PCR Kit or QuantiNova Probe RT-PCR Kit (Qiagen). Primers used for assays were obtained from Eurofins MWG Operon. TSPO1 Fw 5′-CTTTGGTGCCCGACAAATGG-3′ Rv 5′-CTGACCAGCAGGAGATCCAC-3′. Relative quantification of fold change was performed comparing Ct values from individual samples by applying the Pfaffl method [38]. Results were normalized for each of the samples by the expression of the TATA box binding protein (Prime Time® qPCR Assay, Hs.PT.58.19838260; Integrated DNA Technology, Leuven, Belgium).

### 4.10. ATP Measurement

Intracellular ATP was measured by quantitative luminometry using firefly luciferase (EC 1.13.12.7, Sigma-Aldrich), which catalyzes the oxidation of D-luciferin (Life Technologies, Dardilly, France) in the presence of ATP to produce light. Briefly, 3 × 10^5^ cells were frozen and resuspended in 150 μL of water. Then, 5 µL of this lysate was added to 45 µL of modified Krebs buffer (137 mM NaCl, 2.7 mM KCl, 1.5 mM KH_2_PO_4_, 4.72 mM Na_2_HPO_4_, 1.32 mM CaCl_2_, 1.91 mM MgSO_4_, and 5 mM glucose, pH 7.4, 300 mOsm) containing 0.01 µM luciferase, 0.2 mM D-luciferin, and 0.1 mg/mL of Coenzyme A, directly in the assay chamber of a custom-built luminometer as previously described [39], to determine the light intensity. Calibration of the luminometric signal was performed at the end of each measurement with ATP serial dilution from 1 to 16 µM.

### 4.11. ROS Measurement

Cells were stained with CellROX Deep Red Reagent (Thermo Fisher Scientific, C10422; 5 μM) for total ROS staining or with Dihydroethidium (DHE, Sigma Aldrich, D7008; 50 μM) for 30 min at 37 °C. After washing, cells were subsequently analyzed by flow cytometry.

### 4.12. Statistical Analysis

Each experiment was repeated at least 3 times, with a satisfactory correlation between the results of individual experiments. Statistical analyses were performed using Graph Pad Prism 7.0 (Graph Pad Software, San Diego, CA, USA). Data were evaluated using the unpaired t-test and all comparisons with a *p* value less than 0.05 (*p* < 0.05) were considered statistically significant. *p* < 0.0001, *p* < 0.001, *p* < 0.01, and *p* < 0.05 are indicated with four, three, two, or one star(s), respectively. The data are expressed as the mean ± the standard error of the mean (SEM).

## Supplementary Materials

The following are available online at https://www.mdpi.com/1422-0067/21/23/9066/s1.

## Abbreviations

ANT	Adenine Nucleotide Carrier
ATP	Adenosine Triphosphate
BasoE	Basophilic erythroblast
BSA	Bovine serum albumin
CBMCs	Cord blood mononuclear cells
CTRL	Control cells
DMEM	Dulbecco’s modified Eagle’s medium
ER	Endoplasmic reticulum
GPA	Glycophorin A
HBS	Hank buffer saline
IMM	Inner mitochondrial membrane
MGG	May-Grünwald Giemsa
MOI	Multiplicity of infection
OMM	Outer mitochondrial membrane
OrthoE	Orthochromatic erythroblast
PBS	Phosphate buffer saline
PINK1	PTEN-induced kinase-1
PM	Plasma membrane
PolyE	Polychromatic erythroblast
ProE	Proerythroblast
PS	Phospatidylserine
ROS	Reactive oxygen species
shSCR	Short hairpin RNA scramble
shTSPO1	Short hairpin RNA scramble
shTSPO1	Short hairpin RNA targeting TSPO1
TOM40	Translocase of outer mitochondrial membrane 40 homolog
TSPO	Translocator protein 18 kDa
VDAC	Voltage-dependent anion channel
