# Supplementary material for: Downregulation of Mitochondrial TSPO Inhibits Mitophagy and Reduces Enucleation During Human Terminal Erythropoiesis
Source: Int J Mol Sci. 2020 Nov 28;21(23):9066. doi: 10.3390/ijms21239066 (PMC7730461; doi:10.3390/ijms21239066)
Supplement: Supplementary file 1 [file ijms-21-09066-s001.pdf]

Downregulation of Mitochondrial TSPO Inhibits Mitophagy and Reduces Enucleation During Human Terminal Erythropoiesis

Martina Moras [1,2,3], Claude Hattab [1,2,3], Pedro Gonzalez-Menendez [3,4], Suella Martino [1,2,3], Jerome Larghero [5], Caroline Le Van Kim [1,2,3], Sandrina Kinet [3,4], Naomi Taylor [3,4,6], Sophie D. Lefevre [1,2,3,†] and Mariano A. Ostuni [1,2,3,*,†]

[1] Université de Paris, UMR_S1134, BIGR, Inserm, F-75015 Paris, France; martina.moras@inserm.fr (M.M.); chattab@ints.fr (C.H.); suella.martino@inserm.fr (S.M.); caroline.le-van-kim@inserm.fr (C.L.V.K.); sophie.lefevre@inserm.fr (S.D.L.)
[2] Institut National de Transfusion Sanguine, F-75015 Paris, France
[3] Laboratoire d'Excellence GR-Ex, F-75015 Paris, France; pedro.gonzalez-menendez@igmm.cnrs.fr (P.G.-M.); sandrina.kinet@igmm.cnrs.fr (S.K.); naomi.taylor@igmm.cnrs.fr (N.T.)
[4] Institut de Génétique Moléculaire de Montpellier, University of Montpellier, CNRS, F-34293 Montpellier, France
[5] Unité de Thérapie cellulaire, AP-HP, Hôpital Saint-Louis, F-75010 Paris, France; jerome.larghero@aphp.fr
[6] Pediatric Oncology Branch, Center for Cancer Research (CCR), National Cancer Institute (NCI), National Institutes of Health (NIH), Bethesda, MD 20892, USA
* Correspondence: mariano.ostuni@inserm.fr; Tel.: +33-1-4449-3135
† These authors have equally contributed to this work.

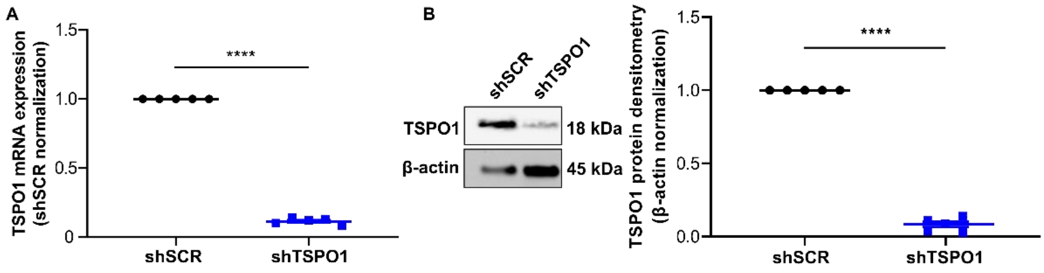

Figure S1. TSPO1 knockdown in erythroid cells at day 10 of ex vivo differentiation. (A) RT-qPCR (n=5) and (B) Western Blot-based quantification (n=5) of TSPO1 levels at day 10 of differentiation to validate the downregulation caused by the shRNA. Levels in shSCR-transduced cells were arbitrarily set at "1". ****p<0.0001.
